# Antiamnesic and Neuroprotective Effects of an Aqueous Extract of *Ziziphus jujuba* Mill. (Rhamnaceae) on Scopolamine-Induced Cognitive Impairments in Rats

**DOI:** 10.1155/2021/5577163

**Published:** 2021-08-10

**Authors:** Etienne Djeuzong, Antoine K. Kandeda, Séfirin Djiogue, Lewale Stéphanie, Danide Nguedia, Florence Ngueguim, Jean P. Djientcheu, Jonas Kouamouo, Théophile Dimo

**Affiliations:** ^1^Department of Pharmacy, University of Montagnes, P.O. Box 208, Banganté, Cameroon; ^2^Department of Animal Biology and Physiology, University of Yaoundé I, P.O. Box 812, Yaoundé, Cameroon

## Abstract

**Background:**

Alzheimer's disease is a neurological condition that affects about 44 million people worldwide. The available treatments target symptoms rather than the underlying causes. *Ziziphus jujuba* (Rhamnaceae) is widely used in traditional Cameroonian medicine to treat diabetes, pain, infections, and dementia. Previous studies reported that *Z. jujuba* aqueous macerate improves working memory impairment, but no study on the antiamnesic effect of a concoction of *Z. jujuba* in rats has been performed. Therefore, this study aimed to assess the antiamnesic and neuroprotective effects of an aqueous extract of *Z. jujuba* on scopolamine-induced cognitive impairments in rats.

**Methods:**

Learning and memory impairments were induced in rats by administering scopolamine (1 mg/kg, i.p.) to 58 rats for 15 days. Rats that developed learning and memory impairments in Morris water maze and Y-maze paradigms were divided into 7 groups (8 rats each) and treated daily for 15 days as follows: the normal control group received distilled water (10 ml/kg, p.o.), the negative control group received distilled water (10 ml/kg, p.o.), positive control groups either received donepezil (1.2 mg/kg, p.o.) or tacrine (10 mg/kg, p.o.), and the three test groups were given the extract (29, 57, and 114 mg/kg, p.o.). At the end of treatments, learning and memory impairments were determined using the same paradigms. Animals were then euthanized, and biochemical parameters of oxidative stress, inflammation, and apoptosis were analyzed in the hippocampus and prefrontal cortex.

**Results:**

On the 4th day of the acquisition phase in the Morris water maze, *Z. jujuba* (29 and 114 mg/kg) reduced (*p* < 0.001) the latency to reach the platform, while in the retention phase, *Z. jujuba* (57 and 114 mg/kg) decreased (*p* < 0.001) the time to reach the platform and increased the time in the target quadrant (*p* < 0.05) compared to control. Surprisingly, the extract failed to affect spontaneous alternations in the *Y*-maze. Furthermore, the extract (29, 57, and 114 mg/kg) reversed (*p* < 0.001) scopolamine-induced oxidative stress, inflammation, and apoptosis. This was supported by the reduction of neuronal alterations in the hippocampus and prefrontal cortex.

**Conclusions:**

Compared to donepezil, a standard drug against Alzheimer's disease, these findings suggest that *Z. jujuba* extract possesses antiamnesic and neuroprotective effects, and these effects are mediated in part through antioxidant, anti-inflammatory, and antiapoptotic activities. These findings help to explain its use in treating psychiatric disorders in Cameroon's folk medicine.

## 1. Introduction

Alzheimer's disease (AD) is the most common form of dementia (60–70% of cases) [1]. It is an irreversible and progressive neurodegenerative disorder of the central nervous system that occurs gradually and leads to memory loss, unusual behavior, and personality changes [1, 2]. According to the World Health Organization (WHO), more than 44 million people worldwide are affected by AD, with 7.7 million new cases annually [3–5]. In Africa, the prevalence of AD is estimated at 5.6%, with 0.96% in Cameroon [6]. At the molecular and cellular levels, AD is characterized by extracellular deposits of beta-4-amyloid protein (BAP), intracellular tangles, cholinergic deficit, extensive neuronal loss, and synaptic changes in the cerebral cortex and hippocampus [7]. Of all types of dysfunctions, BAP plaques and neurofibrillary tangles are the main cytologic hallmarks. BAP seems to initiate the pathogenesis of AD, while neurofibrillary tangles seem to be mainly involved in its progression [2, 3]. BAP deposits are associated with neuronal death via oxidative stress, inflammation, and apoptosis [2]. AD can be modeled in rodents using chemicals, such as D-galactose, glutamate, and scopolamine. Scopolamine, a muscarinic cholinergic receptor antagonist, is used as a pharmacological experimental model for inducing cognitive impairments and memory disorders in animals. Indeed, cognitive behaviors, such as ataxia, anxiety, depression, short-term memory, reference memory, and attention are affected by injections of scopolamine in rodents [1]. Both the hippocampus and prefrontal cortex are susceptible to oxidative stress, and excessive oxidative stress induces memory deficiency by damaging synaptic plasticity and causing inflammation and neuronal cell death [2]. It was found that when scopolamine is injected in rodents, it induces oxidative stress, neuronal inflammation, and apoptosis in the brain [2].

At present, there is no curative treatment for AD [8]. Drug therapies are available, including acetylcholinesterase inhibitors (galantamine, rivastigmine, and donepezil) and the N-methyl-D-aspartic acid receptor antagonist (memantine) [9]. However, these treatments are costly, difficult to access, have significant side effects, and all only relieve symptoms and do not treat the underlying causes [2, 3]. Therefore, there is a need to develop an effective treatment against AD.

Medicinal plants, particularly those used in traditional herbal medicine, constitute a source for discovering new candidates for effective and safe drugs for AD. Among them, *Ziziphus jujuba*, a plant from the Rhamnaceae family, is used in Cameroon, Asia, and India to treat multiple pathologies, including typhoid fever, furuncle, sleep disorders, diarrhea, and pain [10, 11]. In Northern Cameroon, according to claims of traditional healers, all parts of the plant are used to treat otitis, inflammation, cancer, anxiety, rickets, typhoid fever, and anorexia; the seeds are used as dewormers [12] and the leaves for treating cases of dementia [12]. In recent years, research on *Z. jujuba* fruits, seeds, and leaves has revealed anti-inflammatory [13] and neuroprotective activities [14]. The leaves exhibited anti-inflammatory [15], antifungal, anticancer, antifertility, antibacterial, anxiolytic, sedative, and antioxidant properties [16–18]. Pharmacological studies on the antiamnesic effect of *Z. jujuba* reported that the seed has a protective effect on spatial memory impairments in rats [19–21]. According to these authors, this effect was likely mediated by cholinergic blockade [22]. In addition, a hydroethanolic extract of *Z. jujuba* has been shown to ameliorate cognitive decline and seizures in an experimental model of epilepsy in rats [20]. A toxicity study on the leaves of *Z. jujuba* revealed no significant toxicities [23], though toxic elements are found in trace amounts in the whole plant [24]. Phytochemical analysis of the seeds, leaves, and stem bark of *Z. jujuba* indicated alkaloids, flavonoids, tannins, saponins, and polyphenols [25]. Moreover, the HPLC fingerprint of *Z. jujuba* leaf extracts identified the presence of major specific constituents including (‒)-catechin, traumatic acid, quercetin-3-O-robinobioside, rutin, and quercetin-3-O-*α*-L-arabinosyl-(1⟶2)-*α*-L-rhamnoside, with a total of nine flavonoids identified [26]. Furthermore, GC/MS analysis of the ethanol extract of *Z. jujuba* seeds revealed the existence of 20 components, mainly 13-heptadecyn-1-ol (12.95%), 7-ethyl-4-decen-6-one (9.73%), lineoleoyl chloride (8.54%), linoleic acid (6.37%), 2,5-octadecadiynoic acid, methyl ester (5.57%), and palatinol A (4.81%) [27]. Numerous specific chemicals present in the extract have been identified, some of which have been demonstrated to possess anti-inflammatory, antioxidant, and antimalarial activities [26].

To date, there is no solid evidence for the antiamnesic and neuroprotective effects of extracts from *Z. jujuba* leaves, one of the most promising therapeutic properties of the extract. Therefore, this study was undertaken to investigate the antiamnesic and neuroprotective effects of *Z. jujuba* aqueous extract on scopolamine-induced cognitive impairments in rats, using Morris water maze and Y-maze paradigms. Besides, biochemical assays for antioxidant, anti-inflammatory, and antiapoptotic effects and their mechanisms were explored.

## 2. Materials and Methods

### 2.1. Plant Collection and Extraction

The leaves of *Z. jujuba* were harvested in Mokolo (far north region of Cameroon) at global position system coordinates 10.7425° north and 13.8042° east and identified at the National Herbarium of Cameroon (HNC) (database of herbarium index: http://sweetgum.nybg.org/science/ih/herbarium-list) by Mr. Ngansop Eric in comparison to the specimen deposited under the voucher number 14446/HNC/Cam. The plant name has also been checked on http://www.theplantlist.org. The extract of *Z. jujuba* was prepared according to the traditional healers' method. Briefly, fresh leaves of *Z. jujuba* were dried in the shade and crushed into powder. The powder (75 g) was then boiled in 1.5 l of distilled water for 20 min, giving a filtered mixture. The filtrate was finally dried at 50°C to a dry extract (10.8 g), yielding 14.4% (*w/w*). Stock solutions of 2.9 mg/ml, 5.7 mg/ml, and 11.4 mg/ml concentrations were prepared for administration to animals.

### 2.2. Drugs and Chemicals

Donepezil tablets were purchased from U.C.B. Pharma SA Braine-l'Alleud (Belgium) and tacrine hydrochloride capsules from Shionogi. Inc. (Japan). Scopolamine hydrobromide, trichloroacetic acid, ketamine, thiobarbituric acid, Ellman reagent, Griess reagent, adrenaline, and formalin were purchased from Sigma Chemical Co., St. Louis (United States), while diazepam was purchased from Roche (Switzerland). Donepezil, tacrine, and scopolamine were dissolved in distilled water. All solutions were administered *per os* (p.o.) at a volume of 10 ml/kg body weight, except for scopolamine, ketamine, and diazepam, administered intraperitoneally (i.p.).

### 2.3. Animals and Housing Conditions

Animals were male Wistar rats, 6 to 8 weeks old, weighing between 120 and 140 g. Rats were raised in the animal facilities of the Laboratory of Animal Physiology (University of Yaoundé I, Cameroon) under standard light (12-hour day/night cycle), temperature (24 ± 2°C), and humidity (45 ± 3°C) with access to standard animal diet and tap water *ad libitum*. The animals were housed in groups of 5 rats per cage (40 cm × 40 cm). Animal procedures were carried out following the guidelines of the Institutional Ethics Committee of the Cameroon Ministry of Scientific Research and Technological Innovation (Reg. no. FWA-IRD 0001954, 04/09/2006), which adopted the guidelines of the European Union on Animal Care (C.E.E. Council 86/609). Euthanasia of animals was performed according to the American Veterinary Medical Association (AVMA) guidelines for the euthanasia of animals (2020). All animal studies, including allocating animals to experimental groups, experimental dosages, outcomes, and statistical methods, are detailed and carried out according to ARRIVE guidelines 2.0 (http://www.nc3rs.org.uk/page.asp?id=1357). For behavioral experiments, animals were randomly selected.

### 2.4. Experimental Design and Treatment

To induce learning and memory impairments, 58 rats were randomly divided into two groups and treated for 15 days as follows:

- A scopolamine group of 50 rats was treated with scopolamine (1 mg/kg, i.p.)
- A normal control group of 8 rats was given distilled water (10 ml/kg, i.p.)

At the end of the treatments, learning and memory impairments were assessed with the Morris water maze and Y-maze paradigms. Rats that developed learning and memory impairments were selected for further studies. These rats were divided into 7 groups of 8 rats each and treated as follows:

- A negative control group that received distilled water (10 mg/kg, p.o.)
- Two positive control groups that received donepezil (1.2 mg/kg, p.o.) or tacrine (10 mg/kg, p.o.)
- Three test groups that received the aqueous extract of *Z. jujuba* at the doses of 29, 57, and 114 mg/kg, respectively, p.o.

**Figure 1:**
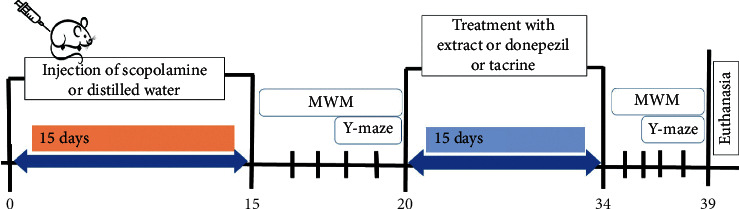
Schematic diagram of the experimental procedure. MWM : Morris water maze.

A normal control group was added as the 7^th^ group (*n* = 7) and treated with distilled water (10 ml/kg, p.o.). All rats were administered the test agents for 15 additional days, and then, cognitive decline or its improvement was assessed with the same water maze and Y-maze paradigms. After completion of the behavioral procedures, rats were sacrificed and brains were removed for analysis of biochemical markers (*n* = 5) and histological assays (*n* = 2) (Figure 1).

### 2.5. Behavioural Studies

#### 2.5.1. Morris Water Maze Test

Spatial learning and long-term memory impairments were assessed using the Morris water maze test [3] with a circular tank of 150 cm diameter and 60 cm height filled to 40 cm with water at 25°C. The tank was divided into four equal quadrants with a white refuge platform of 8 cm diameter and 30 cm height placed in the center of one of the quadrants 1 cm below the water surface. The pool was located in a room with multiple visual cues. On the 1st day of the test, i.e., during the habituation phase, each rat was acclimated for 60 s without the platform. Each rat accomplished three-block sessions with intervals of 30 min between sessions; each block consisted of four successive trials with 60 s duration. On each trial, rats were randomly released into the water from one of the four quadrants facing the maze wall [4, 5]. The acquisition phase started on the 2^nd^ day with the refuge platform and continued for 4 days with three sessions per day. The water was clouded by adding liquid milk so that the platform was invisible from the water surface. The session time for each animal to find the platform was 120 s, and the time interval between sessions was 5 min. When an animal found the platform, it was allowed to stay for 15 s. During each session of the acquisition phase, the latency time to find the platform was recorded for each animal. The effectiveness of learning was then assessed in the retention phase on the 6^th^ day. During this phase, which lasted 120 s, the platform was removed from the tank. Thus, the latency time to find the proper quadrant where the platform was previously and the time spent in this compartment were recorded [6].

#### 2.5.2. Y-Maze Test

The Y-maze test was used to assess working memory in animals by recording spontaneous alternations [7]. The maze used was a wooden device with three identical branches (40 cm long × 35 cm high × 12 cm wide) separated by an angle of 120°. The walls of each arm were decorated with different patterns to differentiate them and labeled as A, B, and C. Rats were individually placed at the end of a maze's branch for free exploration [8]. During 5 min, the number of entries in each arm of the maze was recorded. After each session, the device was cleaned with 10% ethanol to avoid odors. A spontaneous alternation was defined as three successive entries in three different arms, for example, ABC, CAB, or BCA. The percentage of SA was used as an index of working memory and calculated according to the following formula: [(number of SA)/(total number of arms visited −2)]^*∗*^100.

### 2.6. Biochemical Analysis

#### 2.6.1. Animal Euthanasia and Preparation of Homogenates

Following the behavioral assessments, animals were immediately euthanized under anesthesia with ketamine (70 mg/kg, i.p.) and diazepam (10 mg/kg, i.p.). The brain of each rat was removed and divided into two hemispheres. The hippocampus and prefrontal cortex were isolated from one hemisphere, washed in 0.9% NaCl, and blotted to dryness. They were then weighed and homogenized in Tris-HCl buffer (50 mM, pH 7.4) at a ratio of 20% (*w/v*). After centrifugation at 3000 ×g at 4°C for 25 min, the supernatant was collected into tubes and stored at −20°C for further neurochemical evaluation. The other hemisphere of the brain was fixed in 4% formalin for later histological analysis.

#### 2.6.2. Reduced Tissue Glutathione Concentration

Ellman reagent (1.5 ml) was added to tubes containing 100 *μ*l of homogenate or Tris-buffer (50 mM HCl, 150 mM KCl, pH 7.4) as control. The tubes were shaken for 60 min at room temperature (25 ± 1°C), and then, absorbance at 412 nm was read against the control. The concentration of reduced glutathione (GSH) was expressed in *μ*mol/g of tissue proteins [9].

#### 2.6.3. Malondialdehyde Concentration

The malondialdehyde (MDA) assay was carried out according to the method described by Wilbur et al. [10]. Briefly, 250 *μ*l of homogenate was added to the test tubes and 250 *μ*l of Tris-buffer (50 mM HCl; 150 mM KCl; pH 7.4) to the blank tube. Then, 125 *μ*l of 20% trichloroacetic acid and 250 *μ*l of 0.67% thiobarbituric acid were added to each tube, and the tubes were incubated for 10 min at 90°C. They were then cooled and centrifuged at 3000 ×g for 15 min at room temperature (25 ± 1°C). The supernatant was removed, and the absorbance at 530 nm was read against the blank. The concentration of MDA was expressed in mmol/g of tissue protein.

#### 2.6.4. Superoxide Dismutase Activity

The activity of SOD was determined according to the method of Misra and Fridovish [11]. In the blank tube, 1666 *μ*l of carbonate buffer (50 mM, pH 10.2) was added, and 134 *μ*l of homogenate was added to each sample tube. The reaction was started by adding 200 *μ*l of 0.3 mM adrenaline solution. After fast inversion for mixing, the absorbance at 480 nm was read after 20 and 80 s. The specific activity of SOD was expressed in units of SOD/min/g of tissue protein.

#### 2.6.5. Nitrite Concentration

According to the method of Grand et al. [12], the determination of nitrite concentration was carried out. Nitric oxide is a short-half-life compound that is rapidly converted to stable end products, which comprise nitrate (NO3−) and nitrite (NO2−). The conversion of nitrate into nitrite is followed by color development in the presence of 0.1% N-(1-naphthyl) ethylenediamine dihydrochloride, 1% sulfanilamide, and 2.5% phosphoric acid in an acid medium (Griess reagent). Briefly, 200 *μ*l of homogenate and 200 *μ*l of Griess reagent were introduced into test tubes. The solutions were then mixed, and the absorbance was read at 570 nm after 10 min against the blank. Furthermore, a standard curve was established with a set of serial dilutions of nitrite. The obtained equation was used to calculate the unknown sample concentration. The nitrite concentration in the homogenate was expressed in mmol/g of protein in the tissue [13].

#### 2.6.6. Total Protein Concentration

Total protein concentration in the extract was carried out according to the method of Gornall et al. [14], with the protein concentration expressed in µg/ml.

#### 2.6.7. Proinflammatory Markers Concentration

The concentrations of TNF-*α*, IL1-*β*, and IL-6 were determined by Enzyme-Linked Immunosorbent Assay (ELISA) using the Quantikine kit (R and D Systems, Inc., Minneapolis, USA). The concentration of these cytokines was expressed in pg/ml.

#### 2.6.8. Apoptosis Marker Concentration

Quantification of caspases 3 and 9 was carried out by ELISA using the Novus Biologicals kit (R and D Systems, Inc., Minneapolis, USA). The concentration of these apoptosis markers was expressed in ng/ml.

### 2.7. Histopathological Analysis of Brain Tissues

The histological analysis included fixing, cutting, dehydration, inclusion, cutting, coloring, mounting, and observation. The stained and mounted slides were observed at 250X magnification using a Scientico STM-50 optical microscope (HSIDC Industrial Estate, Haryana, India) equipped with a Celestron 44421 digital camera connected to a computer (HP Pavilion, g series). Furthermore, the density of neurons in CA1 and CA3 layers of the hippocampus was determined by counting the number of neurons per 400 *μ*m^2^.

### 2.8. Qualitative Phytochemical Screening

The method of Odebiyi and Sofowora [15] was used to screen flavonoids, alkaloids, saponins, tannins, anthraquinones, triterpenes, and polyphenols, while reducing sugars were ascertained using the method of Harbone [16]. The following reagents were used: flavonoids (NaOH and HCl), alkaloids (H_2_SO_4_^−^and Meyer's reagent), saponins (DMSO, frothing test), polyphenols (DMSO, K3F (CN)6), tannins (DMSO, ferric chloride), reducing sugar (Lieberman's test), and triterpenes (H2SO4^−^ and acetic anhydride).

### 2.9. Quantitative Phytochemical Screening

The concentrations of total phenols, total flavonoids, condensed tannins, total alkaloids, and saponins were determined as previously described by Hatami [17], Dehpour et al. [18], Ba [19], Gracelin [20], and Gracelin [20], respectively.

### 2.10. Statistics

Statistical analyses of the values obtained were carried out using GraphPad Prism version 7.1. (San Diego, CA, USA). Results were expressed as the mean ± standard error of the mean (SEM). Data analyzed were first assessed for normality and sphericity using Shapiro–Wilk and Mauchly's tests, respectively. These tests confirmed that data were normally distributed, but some of them violated the assumption of sphericity. Therefore, the Greenhouse–Geisser test was used for correction. Repeated one-way ANOVA followed by Tukey's *post hoc* test was used to analyze data from the number of spontaneous alternations in the Y-maze test and oxidative stress, proinflammatory cytokines, and apoptosis markers. Repeated two-way ANOVA followed by the Bonferroni *post hoc* test was used to analyze data from the Morris water maze test. At *p* < 0.05, the difference was considered significant.

## 3. Results

### 3.1. Effect of *Z. jujuba* Extract on the Acquisition Phase in the Morris Water Maze

The effect of the extract of *Z. jujuba* during the acquisition phase is shown in Table 1. On the 4th day of the acquisition phase, scopolamine significantly (*p* < 0.001) induced learning deficits in the negative control group when compared to the normal control group (Table 1). In contrast, the extract of *Z. jujuba* (29 and 114 mg/kg) showed the highest (*p* < 0.001) decrease in the latency to reach the platform (Table 1). The effect of the extract was of similar magnitude to that of donepezil, a standard drug against AD known to have this effect (Table 1).

### 3.2. Effect of *Z. jujuba* Extract on the Retention Phase in the Morris Water Maze

**Figure 2:**
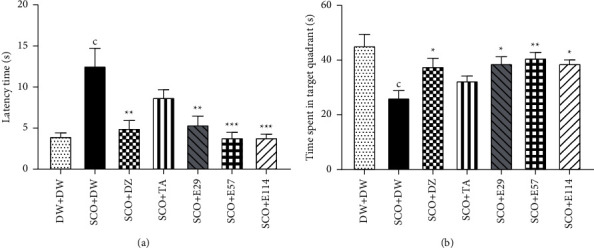
Effect *Z. jujuba* extract on the latency time to reach the platform (a) and the time spent in the target quadrant (b) during the retention phase in the Morris water maze.

The effect of the extract of *Z. jujuba* during the retention phase is shown in Figure 2. During this retention phase, administration of scopolamine in the negative control group significantly (*p* < 0.001) increased the latency to reach the platform when compared to the normal control group (Figure 2(a)). However, the extract of *Z. jujuba* significantly (*p* < 0.001) decreased the latency to reach the platform at all concentrations tested. The effect of the extract was similar to that of donepezil, whereas the decrease by tacrine was not significant (Figure 2(a)).

Also, during this phase, administration of scopolamine in the negative control group significantly (*p* < 0.001) decreased the time spent in the target quadrant when compared to the normal control group (Figure 2(b)). In contrast, the extract at the dose of 57 mg/kg showed the highest (*p* < 0.01) increase in the time spent in the target quadrant (40.9 ± 2.0 s) (Figure 2(b)). This effect of the extract was comparable to that of donepezil, which increased this time to 38.7 ± 1.9 s (*p* < 0.05) (Figure 2(b)).

Each bar represents the average ± SEM; *n* = 7. ^c^*p* < 0.001*vs.* normal control. ^*∗*^*p* < 0.05; ^*∗∗*^*p* < 0.01; ^*∗∗∗*^*p* < 0.001*vs.* negative control. DW: distilled water (10 ml/kg); SCO: scopolamine (1 mg/kg); DZ: donepezil (1.2 mg/kg); TA: tacrine (10 mg/kg); and E29, E57, and E114: aqueous extract of *Z. jujuba* at respective doses of 29, 57, and 114 mg/kg. DW + DW: normal control group; SCO + DW: negative control group; SCO + DZ: positive control group treated with donepezil; SCO + TA: positive control group with tacrine; and SCO + E29-E114: test groups treated with *Z. jujuba* extract.

### 3.3. Effect of *Z. jujuba* Extract on the Spontaneous Alternations in the Y-Maze

**Figure 3:**
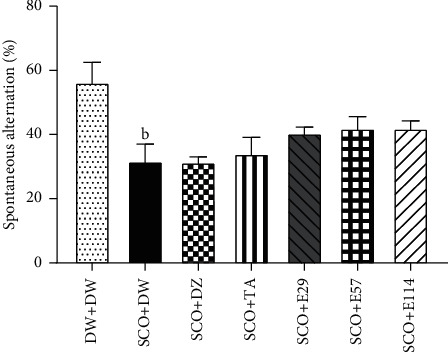
Effect of *Z. jujuba* extract on the percentage of spontaneous alternation in the Y-maze.

The Y-maze test results are presented in Figure 3. A significant (*p* < 0.01) reduction was observed in the number of spontaneous alternations between the negative control group and the normal control group. The extract at all doses, as well as donepezil or tacrine, showed a nonsignificant (*p* > 0.05) increase in this parameter (Figure 3).

Each bar represents the average ± SEM; *n* = 7. ^b^*p* < 0.01*vs.* normal control. DW: distilled water (10 ml/kg); SCO: scopolamine (1 mg/kg); DZ: donepezil (1.2 mg/kg); TA: tacrine (10 mg/kg); and E29, E57, and E114: aqueous extract of *Z. jujuba* at respective doses of 29, 57, and 114 mg/kg. DW + DW: normal control group; SCO + DW: negative control group; SCO + DZ: positive control group treated with donepezil; SCO + TA: positive control group treated with tacrine; and SCO + E29-E114: test groups treated with *Z. jujuba* extract.

### 3.4. Effect of Z*. jujub*a Extract on Some Oxidative Stress Markers in the Hippocampus and Prefrontal Cortex

The effect of the extract of *Z. jujuba* on some oxidative stress markers in the hippocampus and prefrontal cortex is shown in Tables 2 and 3. In the hippocampus and prefrontal cortex, there was a significant (*p* < 0.001) decrease in the concentration of GSH between the negative control group and the normal control group (Tables 2 and 3). However, in the hippocampus, the extract at doses of 29 and 114 mg/kg, as well as tacrine, showed a moderate (*p* < 0.05) increase in the concentration of GSH (77.59%, 72.04%, and 127.62%, respectively), while in the prefrontal cortex, the extract (29 mg/kg) and tacrine showed a significant (*p* < 0.001) increase in the concentration of GSH (264.57% and 209.91%, respectively) (Tables 2 and 3). Tables 2 and 3 show that, in the hippocampus and prefrontal cortex, there was a significant (*p* < 0.001) increase in the concentration of MDA between the negative control group and the normal control group (Tables 2 and 3). In contrast, the extract at all doses, donepezil, and tacrine significantly (*p* < 0.001) decreased the MDA concentration in the hippocampus, whereas in the prefrontal cortex, the extract (114 mg/kg) showed the highest (*p* < 0.01) reduction in this parameter (Tables 2 and 3). The effect of the extract was similar to that of donepezil (*p* < 0.05) or tacrine (*p* < 0.01) (Tables 2 and 3).

In the hippocampus and prefrontal cortex, the activity of SOD significantly (*p* < 0.001) increased between the negative control group and the normal control group (Tables 2 and 3). The extract at all doses, as well as donepezil or tacrine, significantly (*p* < 0.001) reduced this activity (Tables 2 and 3) in both organs. In the hippocampus and prefrontal cortex, the concentration of nitrite significantly (*p* < 0.001) increased in the negative control group when compared to the normal control group (Tables 2 and 3). The extract at the doses of 29 and 114 mg/kg, donepezil, and tacrine showed the most remarkable (*p* < 0.001) decrease in the concentration of nitrite in both organs (Tables 2 and 3).

In the hippocampus and prefrontal cortex, administration of scopolamine caused a significant (*p* < 0.001) decrease in the concentration of total protein between the negative control group and the normal control group (Tables 2 and 3). However, the extract (29 and 144 mg/kg), donepezil, and tacrine significantly (*p* < 0.001) increased this concentration in the two organs (Tables 2 and 3).

### 3.5. Effect of *Z. jujuba* Extract on Some Proinflammatory Markers in the Hippocampus and Prefrontal Cortex

**Figure 4:**
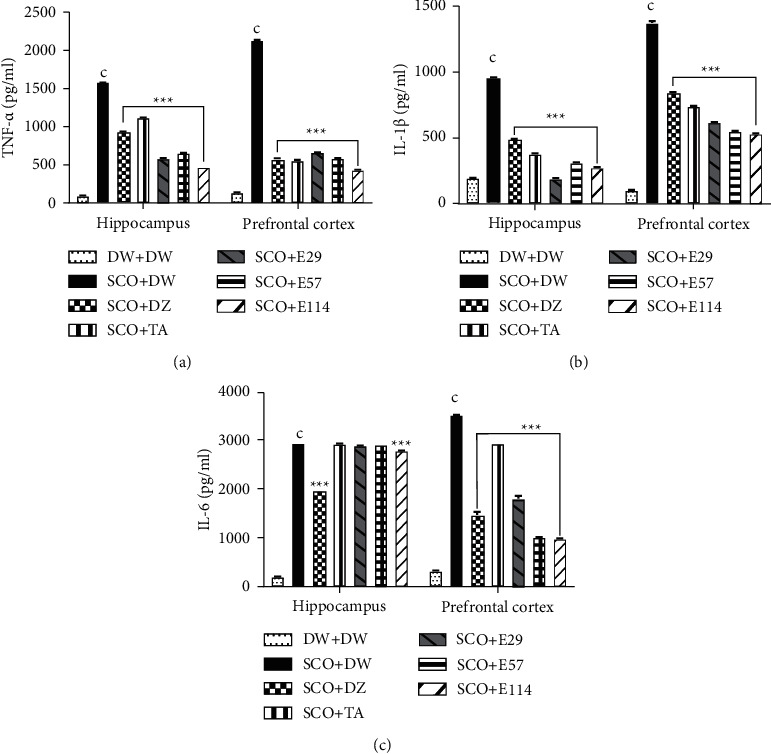
Effect of *Z. jujuba* extract on some proinflammatory markers in the hippocampus and prefrontal cortex. Each value represents the average ± SEM; *n* = 7. ^c^*p* < 0.01*vs.* normal control. ^*∗∗∗*^*p* < 0.001*vs.* negative control. DW: distilled water (10 ml/kg); SCO: scopolamine (1 mg/kg); DZ: donepezil (1.2 mg/kg); TA: tacrine (10 mg/kg); and E29, E57, and E114: aqueous extract of *Z. jujuba* at respective doses of 29, 57, and 114 mg/kg. DW + DW: normal control group; SCO + DW: negative control group; SCO + DZ: positive control group treated with donepezil; SCO + TA: positive control group treated with tacrine; and SCO + E29-E114: test groups treated with *Z. jujuba* extract.

The effect of the extract of *Z. jujuba* on some proinflammatory markers in the hippocampus and prefrontal cortex is shown in Figure 4. The negative control group showed a significant (*p* < 0.001) increase in the concentration of TNF-*α* in the hippocampus and prefrontal cortex (158.4 ± 1.3 pg/ml and 2148.9 ± 1.7 pg/ml, respectively) when compared to the normal control group (Figure 4(a)). The extract (114 mg/kg) showed the highest (*p* < 0.001) reduction of this concentration in the hippocampus and prefrontal cortex (62.47% and 78.96%, respectively) (Figure 4(a)). Similarly, donepezil and tacrine significantly (*p* < 0.001) decreased the TNF-*α* concentration in the hippocampus (42.32% and 28.76%, respectively) and prefrontal cortex (78.06% and 73.16%, respectively) (Figure 4(a)).The negative control group showed a significant (*p* < 0.001) increased IL-1 *β* concentration in the hippocampus (112.1 ± 0.8 pg/ml) and prefrontal cortex (1281.3 ± 1.1 pg/ml) when compared to the normal control group (Figure 4(b)). The extract (29 mg/kg), donepezil, and tacrine significantly (*p* < 0.001) decreased the IL-1*β* concentration in the hippocampus and prefrontal cortex to79.69%, 55.66%, and 61.04%, respectively (Figure 4(b)), while in the prefrontal cortex, the extract (114 mg/kg), donepezil, and tacrine significantly (*p* < 0.001) increased the IL-1*β* concentration to 60.12%, 36.04%, and 45.42%, respectively (Figure 4(b)).The administration of scopolamine in the negative control group significantly (*p* < 0.001) increased the concentration of IL-6 in the hippocampus and prefrontal cortex to 346.1 ± 8.7 pg/ml and 3526.3 ± 3.4 pg/ml, respectively, when compared to the negative control group (Figure 4(c)). The extract at all doses, as well as donepezil or tacrine, significantly (*p* < 0.001) decreased the concentration of IL-6 in the hippocampus and prefrontal cortex (Figure 4(c)).

### 3.6. Effect of *Z. jujuba* Extract on Some Apoptosis Markers in the Hippocampus and Prefrontal Cortex

**Figure 5:**
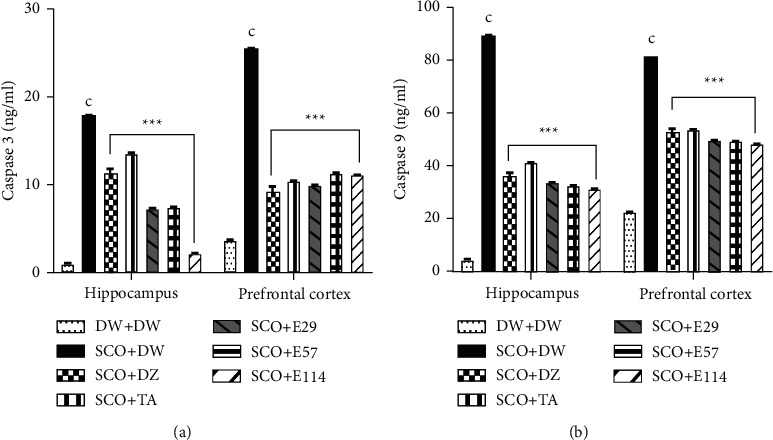
Effect of *Z. jujuba* extract on some apoptosis markers in the hippocampus and prefrontal cortex. Each value represents the average ± SEM; *n* = 7. ^c^*p* < 0.01*vs.* normal control. ^*∗∗∗*^*p* < 0.001*vs.* negative control. DW: distilled water (10 ml/kg); SCO: scopolamine (1 mg/kg); DZ: donepezil (1.2 mg/kg); TA: tacrine (10 mg/kg); and E29, E57, and E114: aqueous extract of *Z. jujuba* at respective doses of 29, 57, and 114 mg/kg. DW + DW: normal control group; SCO + DW: negative control group; SCO + DZ: positive control group treated with donepezil; SCO + TA: positive control group treated with tacrine; and SCO + E29-E114: test groups treated with *Z. jujuba* extract.

The effect of the extract *Z. jujuba* on some apoptosis markers is shown in Figure 5. In the negative control group, the concentration of caspase 3 significantly (*p* < 0.001) increased in the hippocampus and prefrontal cortex (18.5 ± 0.1 ng/ml and 25.4 ± 0.1 ng/ml, respectively) when compared to the normal control group (Figure 5(a)). In the hippocampus, the extract of *Z. jujuba* (144 mg/kg), donepezil, and tacrine significantly (*p* < 0.001) decreased this concentration to 87.94%, 39.28%, and 24.03%, respectively, while in the prefrontal cortex, the extract (29 mg/kg), donepezil, and tacrine significantly (*p* < 0.001) decreased this concentration to 60.95%, 66.23%, and 59.05%, respectively (Figure 5(a)). In the negative control group, the concentration of caspase 9 significantly (*p* < 0.001) increased in the hippocampus and prefrontal cortex (84.7 ± 0.1 ng/ml and 78.6 ± 0.2 ng/ml, respectively) when compared to the normal control group (Figure 5(b)). In contrast, the extract (114 mg/kg), donepezil, and tacrine significantly decreased (*p* < 0.001) the concentration of caspase 9 in the hippocampus (64.67%, 59.09%, and 53.43%, respectively) and prefrontal cortex (40.34%, 29.88%, and 33.58%, respectively) (Figure 5(b)).

### 3.7. Effect of *Z. jujuba* Extract on Neuronal Alterations in the Hippocampus and Prefrontal Cortex

**Figure 6:**
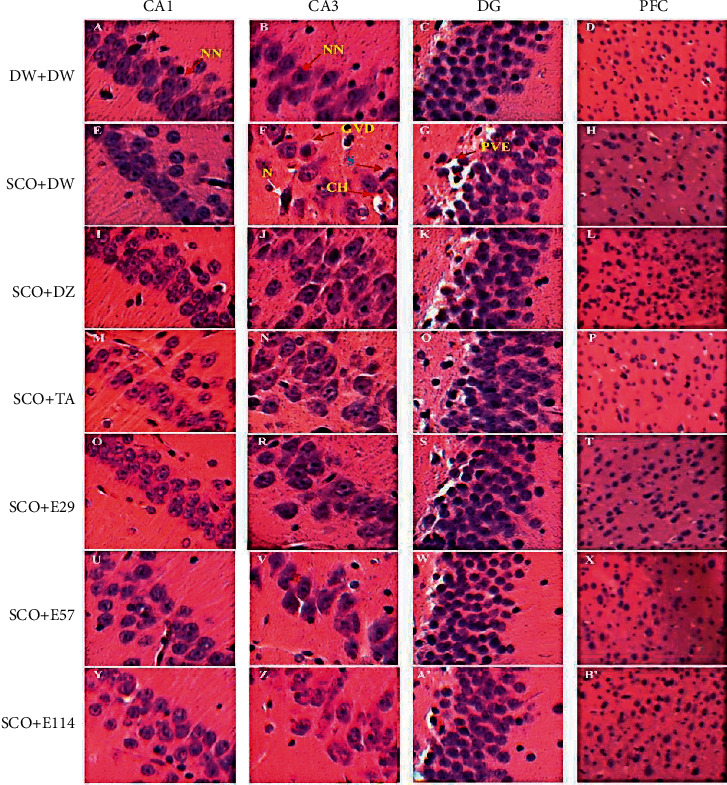
Photographs of the hippocampal and prefrontal cortex sections after hematoxylin and eosin staining (250X). *Hippocampus* (CA1, CA3, and DG); PFC: prefrontal cortex; CA1 and 3: *Cornu ammonis* 1 et 3; DG: dentate gyrus; N: neuron; NN: normal neuron; GVD: granulovacuolar degeneration; S spongiosis; CH: chromatolysis; PVE: perivascular edema; DW: distilled water (10 ml/kg); SCO: scopolamine (1 mg/kg); DZ: donepezil (1.2 mg/kg); TA: tacrine (10 mg/kg); and E29, E57, and E114: aqueous extract of *Z. jujuba* at respective doses of 29, 57, and 114 mg/kg. DW + DW: normal control group; SCO + DW: negative control group; SCO + DZ: positive control group treated with donepezil; SCO + TA: positive control group treated with tacrine; and SCO + E29-E114: test groups treated with the extract of *Z. jujuba*.

The microarchitecture of the hippocampus of the normal control group showed intact neurons in CA1 and CA3 layers (Figure 6(A and B)) and regular thickness in the dentate gyrus, while in the prefrontal cortex, it was observed a normal density of neurons (Figure 6 (D)). Equally, the hippocampus of rats treated with the extract (29 and 57 mg/kg), as well as donepezil, showed a regular thickness of CA1 and CA3 layers (Figure 6 (Q, *R*, U, and V)) and decreased perivascular edema (Figure 6 (S and W)), while in the prefrontal cortex, it was observed a normal density of neurons (Figure 6 (T and X)). Compared to the abovementioned treatments, the hippocampus of the negative control group showed a thickness of CA1 and CA3 layers, reduced density of neural cell bodies, spongiosis (S), granulovacuolar degeneration (GVD), and chromatolysis (CH) (Figure 6 (E and 6F)); it was also observed the presence of perivascular edema (PE) in the dentate gyrus (Figure 6 (G)). Furthermore, the prefrontal cortex of this group showed a low density of neurons (Figure 6 (H)).

### 3.8. Effect of *Z. jujuba* Extract on the Density of CA1 and CA3 Neurons in the Hippocampus

Table 4 presents the effect of the extract of *Z. jujuba* on the density of CA1 and CA3 neurons in the hippocampus. The negative control group showed a 45% (*p* < 0.001) and 38% (*p* < 0.05) decrease in the density of CA1 and CA3 neurons, respectively, as compared to the normal control group. Nonetheless, the extract (29 and 57 mg/kg) and donepezil showed the highest (*p* < 0.05 − 0.001) increase in the density of CA1 and CA3 neurons, whereas tacrine only increased (*p* < 0.05) this density in the CA1 layer (Table 4).

### 3.9. Qualitative Phytochemical Analysis of *Z. jujuba* Extract

Qualitative phytochemical screening of the *Z. jujuba* aqueous extract indicated flavonoids, phenols, anthraquinones, coumarins, tannins, triterpenes, anthocyanins, phenols, and reducing sugars, but no significant alkaloids and saponins.

### 3.10. Quantitative Phytochemical Analysis of *Z. jujuba* Extract

Compared to standards, total phenolic compounds (133.22 ± 0.24 mg gallic acid equivalent/g), condensed tannins (20.66 ± 0.21 mg catechin equivalent/g), and flavonoid contents (11.99 ± 0.68 mg rutin equivalent/g) were abundant, while total alkaloids (7.24 ± 0.37%) and saponins (3.66 ± 0.23%) were less abundant.

## 4. Discussion

Alzheimer's disease (AD) is an irreversible, progressive brain disease that destroys memory and thinking skills and, eventually, the ability to perform the simplest tasks [21]. Until now, there is no curative treatment for AD. Thus, natural resources such as medicinal plants have been used to treat various memory disorders such as dementia, AD, and Parkinson's disease for a long time [21]. Previous studies demonstrated that an aqueous macerate of *Z. jujuba* alleviates working memory impairment and restores neurochemical changes in the prefrontal cortex of D-galactose-treated rats [22]. However, the antiamnesic effect of an aqueous extract (concoction) of *Z. jujuba* was not assessed on learning and memory deficits. Therefore, this study investigated the effect of an aqueous extract (concoction) of *Z. jujuba* on scopolamine-induced cognitive impairments in rats using the Morris water maze and Y-maze paradigms. It is well known that memory impairments, especially those focused on episodic memory, are one of the major symptoms of AD [23], and the Morris water maze is commonly used to assess this type of memory impairment in rodents. The Morris water maze is a powerful and sensitive tool for evaluating hippocampal-spatial learning and reference memory. It is also used to identify drugs with antiamnesic properties, i.e., drugs preventing or restoring or reducing memory loss following brain injury [24]. In the present study, the administration of scopolamine for 15 days decreased the latency to find the platform during the acquisition phase (day 1 to day 4). However, on the 4th day of this phase, scopolamine markedly increased this time compared to the normal group, suggesting, thus, impairment of learning functions in rats. Twenty-four hours following the acquisition phase, i.e., during the retention phase (6th day of the experiment), administration of scopolamine increased the latency to find the platform and reduced the time spent in the target quadrant, as expected, suggesting an impairment of the reference memory. These findings corroborate those of Li et al. [25]. Scopolamine induces amnesia by acting as a nonselective agonist of muscarinic acetylcholine receptors of the M1 and M5 subtypes, acetylcholine being a critical neurotransmitter involved in long-term learning and memory processing [26], thus leading to recognition memory (place and identity memory) impairment. Therefore, alteration of the cholinergic system is followed by impairment of recognition memory during the acquisition and retention phases [27, 28]. In contrast, during both phases, the extract of *Z. jujuba* at all the doses and the positive control agent donepezil reversed these alterations, indicating that the extract has improved the remembrance of the platform position and, hence, better long-term spatial learning and memory functions in rats compared to the normal control group. These findings suggest an antiamnesic effect of the extract on long-term spatial learning and memory deficit [25, 29] and agree with those demonstrating that an ethanolic extract of *Z. jujuba* ameliorates cognitive impairments in rodents [25]. The fact that the extract restored learning and memory impairments similarly to donepezil, an inhibitor of acetylcholinesterase activity, suggests a blockade of cholinergic pathways by the extract as well, although the specific molecular components and mechanisms for the effects of the extract remain to be established. These results also indicate a beneficial effect of the extract on long-term learning and memory dysfunctions [25, 29]. Furthermore, bioactive molecules belonging to the classes of flavonoids, alkaloids, and tannins have been shown to improve long-term memory in rodents [30–32]; some of these compounds (flavonoids and tannins) were identified as present in abundance in the extract, revealing that they might be responsible for the antiamnesic properties of the extract. Further studies will be required to isolate bioactive molecules in the extract and study their individual effects on long-term learning and memory impairments.

Working memory impairments are one of the earlier symptoms of AD, for example, causing AD patients to forget the question that they just asked [33]. The prefrontal cortex is a key brain area for working memory, and injury to this brain area may induce deficits in working memory [34]. These patterns can be modeled in rodents by an intraperitoneal administration of scopolamine, which leads to the phosphorylation of tau proteins, the subsequent formation of beta-amyloid protein in the brain, and working memory deficits [34]. The Y-maze paradigm is usually preferred to assess working memory impairments in rodents [34], and it is based on the willingness of rodents to explore new environments [34]. Thus, normal animals prefer to explore a different arm of the maze than the one they visited on their previous entry. In rats receiving scopolamine and distilled water for 15 days, the percentage of spontaneous alternations in maze arm entry decreased, indicating that the animal has forgotten the arm it just visited. These observations suggested that, thus, impairment of working memory was comparable to those obtained in a previous study with the hydromethanolic extract of *Ziziphus mucronata* on scopolamine-induced amnesia in rats [35]. Surprisingly, the extract at all the doses and all standards did not significantly affect the percentage of spontaneous alternations, suggesting that they do not interfere with working memory dysfunctions. Therefore, they do not exert antiamnesic properties on short-term memory impairments [7, 36, 37]. These findings contrast with those obtained in recent studies with the aqueous macerate of *Z. jujuba* on the D-galactose model and reveal that the aqueous macerate is more effective than the concoction of the same plant [22]; maybe the aqueous macerate of *Z. jujuba* contains bioactive molecules that interfere with the process of short-term memory. Further studies on short-term memory impairments should be performed to confirm the obtained results in the present study.

In AD, the deposition of beta-4-amyloid proteins in the brain induces the generation of reactive oxygen species (ROS) [33, 38], and the increase in the level of these molecules is associated with learning and memory impairments through the alteration of proteins, lipids, and DNA in neurons [33, 38]. Besides, a previous study measured antioxidant enzyme activity and peroxide content in the brain tissue of patients with AD. The results revealed that GSH and SOD activities were significantly lower than those in healthy individuals, while MDA levels were markedly increased [39]. In the present study, administration of scopolamine for 15 days increased MDA, SOD, and nitrite levels and decreased those of GSH and total protein, indicating overproduction of free radicals (increased MDA and nitrite), inactivation of detoxification systems (GSH depletion), and consumption of antioxidants (increased SOD activity) in the hippocampus and prefrontal cortex. These results, therefore, suggest increased oxidative stress in the hippocampus and prefrontal cortex [39] and are consistent with those of Isola et al. [40] and Ghasemi et al. [41]. It is well established that oxidative stress is among the first and fundamental mechanisms of cell damage following the administration of scopolamine [3]. Indeed, during scopolamine-induced cognitive impairment, oxidative damage of lipids by lipid peroxidation results in the production of a wide variety of end products, including MDA, which is accepted as a reliable oxidative stress marker of lipid peroxidation in learning and memory deficits [42]. This process also includes the depletion of GSH, an intracellular thiol-based antioxidant present in reduced form, and has an essential role in the cellular defence cascade against oxidative injury; it acts as a cofactor for glutathione peroxidase and prevents the generation of hydroxyl free radicals [42]. All these changes disrupt the normal antioxidative defence ability of brain tissue. However, the administration of the extract at all doses and donepezil or tacrine markedly counteracted the scopolamine-induced increase in SOD, nitrite, and MDA levels compared to the normal control group and suggest either the inhibition of the lipid peroxidation or SOD overproduction stimulation. Moreover, the extract also reversed the depletion of GSH and increased total protein in both brain regions, suggesting direct scavenging of ROS or GSH production stimulation. These findings, therefore, reveal that the extract possesses significant and potentially valuable antioxidant activity, and this activity was more prominent than that of donepezil or tacrine with light antioxidant properties [9]. These results are consistent with several prior studies which demonstrated the antioxidant properties of *Z. jujuba* [43–45] and with those of Yoo et al. [46]. They showed that the neuroprotective effect of a methanolic extract of *Z. jujuba* against ischemic damage in the gerbil was due to its antioxidant properties. Furthermore, the presence of phenols, alkaloids, and flavonoids in the extract, evidenced in this study, may account for its antioxidant properties since several studies have shown that these compounds' cytoprotective and neuroprotective properties are strongly correlated to their antioxidant potential [47, 48]. Taken together, these findings likely explain, at least in part, the antiamnesic and neuroprotective effects of the extract [46].

Reactive oxygen species-caused oxidative stress is considerably correlated with neuroinflammation, which exacerbates neurodegenerative disorders and contributes to the progression of AD [49]. Neuroinflammation is not generally seen as a cause of AD, but rather as a result of AD, in which microglia are overactivated, resulting in the increased production of proinflammatory cytokines TNF-*α*, IL-1*β*, and IL-6, some of the main drivers of neuroinflammation [49]. Similarly, deficiencies in anti-inflammatory mechanisms may contribute to neuroinflammation in AD [50]. A potential mechanism for the increase in these proinflammatory cytokines is through the inhibition of nuclear factor kappa-B (NFKB) [51], a known signal transduction mediator that is mainly involved in the control of the synthesis of proinflammatory cytokines in the cell [50]. Therefore, drugs with antiinflammatory activity could contribute to halting neuronal loss and improving the learning and memory deficits in patients with AD [51].

In the present study, repeated administration of scopolamine to distilled-water-treated rats induced a strong inflammatory response characterized by increased levels of proinflammatory cytokines (TNF-*α*, IL-1*β*, and IL-6) levels in the hippocampus and prefrontal cortex as compared to the normal group and suggested severe inflammation in these brain regions. Following intraperitoneal injection of scopolamine, an inflammatory response occurs mainly in the limbic system, which is characterized by the upregulation of proinflammatory cytokines and the activation of glial cells [52]. Activated glial cells and the overexpression of proinflammatory cytokines have been correlated with the injury of the hippocampus and often prefrontal cortex [52]. The aqueous extract and donepezil or tacrine mitigated the inflammatory processes induced by scopolamine in the hippocampus and prefrontal cortex, suggesting anti-inflammatory properties abundantly reported in the literature [51]. These data corroborate those demonstrated by Kandeda et al. [22] with the aqueous macerate of the same plant on the D-galactose model in rats. The extract's anti-inflammatory properties likely contribute to its antiamnesic effect, although further studies are needed to confirm this mechanism. The anti-inflammatory properties of the extract are also consistent with the neuroprotective effect, given that drugs with anti-inflammatory activities are known to reduce or prevent damage induced by inflammation in the brain [53]. Furthermore, the identified chemical compounds in the extract, including flavonoids, alkaloids, phenols, and tannins, might underlie this activity [15] since these compounds have been shown to possess anti-inflammatory activities in some experimental models of brain diseases [54]. This extract could have therapeutic value in treating learning and memory deficits and various brain diseases associated with neuroinflammation.

Compelling evidence indicates that oxidative stress and inflammation can trigger apoptosis mechanisms as part of AD pathogenesis [55]. Therefore, inhibiting these dysfunctions and the resultant apoptosis may reduce neuronal loss and cognitive impairments. In the present study, scopolamine increased the concentration of caspases 3 and 9, two significant contributors to apoptosis, in both the hippocampus and prefrontal cortex of the negative control group, similar to the results of Demirci et al. [56] with scopolamine-induced dementia in aged rats. Several studies established that scopolamine induces the formation of beta-4-amyloid protein (BAP) in the hippocampus and prefrontal cortex [57], and BAP, via caspases 3, 8, and 9, leads to apoptosis of neuronal cells and contributes to the pathophysiology of AD [58]. In addition, this drug is also responsible for the initiation of apoptosis by promoting the overexpression of the proapoptotic protein, Bax, leading to cell death by increasing caspase activator release in the brain tissues [59, 60]. In the present study, scopolamine increased the levels of caspases 3 and 9 in the hippocampus and prefrontal cortex, confirming, thus, the abovementioned findings. Treatment with the extract at all doses as well as donepezil or tacrine resulted in a significant decrease in the concentration of caspases assessed by ELISA in both the hippocampus and prefrontal cortex, suggesting an alteration of apoptotic mechanisms. These results also indicate that the extract is likely to exhibit antiapoptotic activity, contributing to its neuroprotective effect [13, 61]. These properties might involve the action of some of the most abundant secondary metabolites, such as flavonoids and polyphenols, found in the extract, with antiapoptotic potential [59, 60]. Further studies will be required to identify the specific components of the extract that mediate its effects on caspase expression and the regulatory pathways involved.

In both humans and animals, the prefrontal cortex and hippocampus and their communication have been reported to play an essential role in the encoding and retrieval of diverse memory processes [62]. Consequently, damage in these areas is associated with severe cognitive impairments [62]. Analysis of the histological sections of the scopolamine-treated rats showed an alteration in the structure of CA1 and CA3 areas of the hippocampus of the negative control group. It was also observed the same patterns in the prefrontal cortex. These histological modifications are confirmed by a decrease in the number of neurons of CA1 and CA3. These findings suggest damage in both regions and ensure those obtained in previous studies [62]. However, the extract (29 and 57 mg/kg) or donepezil alleviated structural damage in the hippocampus and prefrontal cortex. These modifications were confirmed by a significant increase in the number of neurons in CA1 and CA3 areas of the hippocampus.

Given that CA1, CA3, and prefrontal cortex regions are involved in the learning and memory process [63], these findings are consistent with the neuroprotective effect of the extract. This neuroprotective effect is likely to be mediated by the antioxidant, anti-inflammatory, and antiapoptotic activities we have demonstrated for the extract. Indeed, several studies reported that oxidative stress, inflammation, and apoptosis are all involved in neuronal alterations or death in the brain [56].

Taken together, this study here suggests that this extract or specific components derived from it could be used to prevent many of the long-term learning and memory dysfunctions and other neurodegenerative processes that occur in patients with AD, epilepsy, and Parkinson's disease. Further studies on isolated molecules from this extract will help establish the exact components and mechanisms involved in the potentially clinically relevant antiamnesic and neuroprotective effects of this natural plant extract.

## 5. Conclusions

These studies document the antiamnesic and neuroprotective effects of *Z. jujuba* aqueous extract on the scopolamine model of AD in rats. Treatment with the aqueous extract protected animals from long-term learning and memory impairments. Analysis of possible modes of action suggests that these effects may be mediated, at least in part, by the antioxidant, anti-inflammatory, and antiapoptotic activities documented for the extract. These effects of the extract were better than those of donepezil, a standard drug against AD. The antiamnesic and neuroprotective effects of the extract shown here support the long-time empirical use of this extract to treat dementia in Cameroonian folk medicine. Furthermore, this extract or its components could be used as an adjunct to treat diseases associated with long-term learning and memory impairments such as AD and related memory disorders.

## Figures and Tables

**Table 1:** Effect *Z. jujuba* extract on the latency to reach the platform during the acquisition phase in the Morris water maze.

Treatment	Day 1 lat. (s)	Day 2 lat. (s)	Day 3 lat. (s)	Day 4 lat. (s)
DW + DW	21.3 ± 4.4	8.3 ± 0.8	6.9 ± 0.5	5.9 ± 0.2
SCO + DW	29.3 ± 3.5	15.0 ± 2.0	14.1 ± 2.2	14.5 ± 2.7^c^
SCO + DZ	24.7 ± 2.2	13.3 ± 2.6	12.8 ± 2.5	6.1 ± 1.1^*∗∗*^
SCO + TA	51.7 ± 4.3	10.6 ± 1.8	9.8 ± 1.5	10.9 ± 1.4
SCO + E29	38.8 ± 2.1	9.8 ± 0.9	6.2 ± 0.8	5.2 ± 0.4^*∗∗∗*^
SCO + E57	36.9 ± 6.7	6.9 ± 0.7	8.8 ± 0.9	7.2 ± 0.7^*∗∗*^
SCO + E114	27.2 ± 3.4	6.7 ± 1.5	7.4 ± 0.6	6.0 ± 0.7^*∗∗∗*^

Each value represents the average ± SEM; *n* = 7. ^c^*p* < 0.001*vs.* normal control. ^*∗∗*^*p* < 0.01; ^*∗∗∗*^*p* < 0.001*vs.* negative control. s: second; lat.: latency time; DW: distilled water (10 ml/kg); SCO: scopolamine (1 mg/kg); DZ: donepezil (1.2 mg/kg); TA: tacrine (10 mg/kg); E29, E57, and E114: aqueous extracts of *Z. jujuba* at respective doses of 29, 57, and 114 mg/kg. DW + DW: normal control group; SCO + DW: negative control group; SCO + DZ: positive control group with donepezil; SCO + TA: positive control group treated with tacrine; SCO + E29-E114: test groups treated with *Z. jujuba* extract.

**Table 2:** Effect of *Z. jujuba* extract on some oxidative stress markers in the hippocampus.

Treatment	Oxidative stress markers in the hippocampus				
	GSH (*μ*mol/g)	MDA (mmol/g)	SOD (unit/min/g)	Nitrite (mmol/g)	Total protein (*μ*g/ml)
DW + DW	690.1 ± 48.3	17.1 ± 1.2	14.4 ± 0.8	144.1 ± 1.7	1751.3 ± 24.1
SCO + DW	316.4 ± 18.9^c^	64.6 ± 3.4^c^	146.1 ± 14.3^c^	532.4 ± 3.4^c^	450.8 ± 13.2^c^
SCO + DZ	322.1 ± 23.5	27.0 ± 1.6^*∗∗∗*^	201.3 ± 19.4^*∗∗∗*^	320.9 ± 3.6^*∗∗∗*^	1289.5 ± 17.8^*∗∗∗*^
SCO + TA	720.1 ± 85.6^*∗*^	40.5 ± 7.4^*∗∗∗*^	743.6 ± 34.9^*∗∗∗*^	409.2 ± 2.2^*∗∗∗*^	1176.2 ± 34.4^*∗∗∗*^
SCO + E29	561.9 ± 30.3^*∗*^	16.3 ± 1.3^*∗∗∗*^	191.1 ± 15.5^*∗∗∗*^	332.8 ± 2.2^*∗∗∗*^	1139.9 ± 39.3^*∗∗∗*^
SCO + E57	319.4 ± 11.5	17.9 ± 1.1^*∗∗∗*^	187.9 ± 10.8^*∗∗∗*^	331.2 ± 3.8^*∗∗∗*^	1352.3 ± 26.4^*∗∗∗*^
SCO + 114	544.3 ± 33.2^*∗*^	28.3 ± 1.8^*∗∗∗*^	260.4 ± 13.2^*∗∗∗*^	327.2 ± 2.6^*∗∗∗*^	1466.3 ± 21.1^*∗∗∗*^

Each value represents the average ± SEM; *n* = 7. ^c^*p* < 0.001*vs.* normal control. ^*∗*^*p* < 0.05; ^*∗∗∗*^*p* < 0.001*vs.* negative control; DW: distilled water (10 ml/kg); SCO: scopolamine (1 mg/kg); DZ: donepezil (1.2 mg/kg); TA: tacrine (10 mg/kg); E29, E57, and E114: aqueous extract of *Z. jujuba* at respective doses of 29, 57, and 114 mg/kg. DW + DW: normal control group; SCO + DW: negative control group; SCO + DZ: positive control group treated with donepezil; SCO + TA: positive control group with tacrine; SCO + E29-E114: test groups treated with *Z. jujuba* extract; GSH: reduced glutathione; MDA: malondialdehyde; SOD: superoxide dismutase.

**Table 3:** Effect of *Z. jujuba* extract on some oxidative stress markers in the prefrontal cortex.

Treatment	Oxidative stress markers in the prefrontal cortex				
	GSH (*μ*mol/g)	MDA (mmol/g)	SOD (unit/min/g)	Nitrite (mmol/g)	Total protein (*μ*g/ml)
DW + DW	1134.1 ± 102.4	4.5 ± 0.5	61.3 ± 5.1	169.6 ± 1.7	2170.9 ± 36.1
SCO + DW	232.6 ± 33.7^c^	13.3 ± 2.3^c^	212.6 ± 373.2^c^	613.6 ± 2.8^c^	331.6 ± 32.2^c^
SCO + DZ	487.3 ± 49.2	7.2 ± 2.6^*∗*^	126.5 ± 16.6^*∗∗∗*^	402.7 ± 2.4^*∗∗∗*^	1202.2 ± 33.9^*∗∗∗*^
SCO + TA	720.9 ± 75.2^*∗∗*^	6.2 ± 1.1^*∗∗*^	59.4 ± 7.3^*∗∗∗*^	530.4 ± 3.2^*∗∗∗*^	1404.1 ± 25.1^*∗∗∗*^
SCO + E29	848.1 ± 87.9^*∗∗∗*^	9.5 ± 0.7	271.8 ± 24.1^*∗∗∗*^	370.4 ± 3.5^*∗∗∗*^	958.6 ± 28.4^*∗∗∗*^
SCO + E57	710.4 ± 79.9^*∗∗*^	9.5 ± 1.2	395.1 ± 48.3^*∗∗∗*^	366.4 ± 2.8^*∗∗∗*^	865.3 ± 26.7^*∗∗∗*^
SCO + 114	538.1 ± 56.9	5.3 ± 0.5^*∗∗*^	321.5 ± 27.5^*∗∗∗*^	425.2 ± 1.1^*∗∗∗*^	683.9 ± 32.4^*∗∗∗*^

Each value represents the average ± SEM; *n* = 7. ^c^*p* < 0.001*vs.* normal control. ^*∗*^*p* < 0.05; ^*∗∗*^*p* < 0.01; ^*∗∗∗*^*p* < 0.001*vs.* negative control; DW: distilled water (10 ml/kg); SCO: scopolamine (1 mg/kg); DZ: donepezil (1.2 mg/kg); TA: tacrine (10 mg/kg); E29, E57, and E114: aqueous extract of *Z. jujuba* at respective doses of 29, 57, and 114 mg/kg. DW + DW: normal control group; SCO + DW: negative control group; SCO + DZ: positive control group with donepezil; SCO + TA: positive control group treated with tacrine; SCO + E29-E114: test groups treated with *Z. jujuba* extract; GSH: reduced glutathione; MDA: malondialdehyde; SOD: superoxide dismutase.

**Table 4:** Effect of *Z. jujuba* extract on the density of CA1 and CA3 neurons in the hippocampus.

	DW + DW	SCO + DW	SCO + DZ	SCO + TA	SCO + E29	SCO + E57	SCO + 114
CA1	110.1 ± 3.1	61.1 ± 1.7^c^	88.6 ± 2.3^*∗∗∗*^	74.1 ± 1.1^*∗*^	91.7 ± 1.2^*∗∗∗*^	85.3 ± 3.7^*∗∗∗*^	78.5 ± 1.5^*∗∗*^
CA3	46.3 ± 1.5	37.5 ± 1.5^a^	44.1 ± 2.4^*∗*^	41.5 ± 1.5	45.5 ± 1.5^*∗*^	45.1 ± 1.1^*∗*^	40.5 ± 0.5

Each value represents the average ± SEM; *n* = 7. ^a^*p* < 0.05; ^c^*p* < 0.001*vs.* normal control. ^*∗*^*p* < 0.05; ^*∗∗*^*p* < 0.01; ^*∗∗∗*^*p* < 0.001*vs.* negative control; DW: distilled water (10 ml/kg); SCO: scopolamine (1 mg/kg); DZ: donepezil (1.2 mg/kg); TA: tacrine (10 mg/kg); E29, E57, and E114: aqueous extract of *Z. jujuba* at respective doses of 29, 57, and 114 mg/kg. DW + DW: normal control group; SCO + DW: negative control group; SCO + DZ: positive control group with donepezil; SCO + TA: positive control group with tacrine; SCO + E29-E114: test groups treated with the extract of *Z. jujuba*; CA1 : *Cornus ammonis* 1; CA3 : *Cornus ammonis* 3.

## Data Availability

The datasets used and/or analyzed during the current study are available from the corresponding author on simple request.
